# Parenthood and Life Satisfaction in Stratified Labor Market: Evidence From Korea

**DOI:** 10.3389/fpubh.2022.874877

**Published:** 2022-06-02

**Authors:** Joonmo Cho, Hanna Jung

**Affiliations:** ^1^School of Economics, Sungkyunkwan University, Seoul, South Korea; ^2^School of Economics, Mokpo National University, Muan-gun, South Korea

**Keywords:** parenthood status, life satisfaction, labor force, employment insecurity, firm size

## Abstract

This study analyzes the effect of parenthood on life satisfaction with a stratified labor market using the Korean Labor and Income Study. For regular female workers at large companies, the decrease in life satisfaction due to parenthood is higher compared to that for men in a similar position due to the high opportunity cost of a career break following childbirth. For men who are non-regular employees at Small and medium-sized enterprises (SMEs), the effect of parenthood on life satisfaction is negative because they are the income earners of the family but earn a relatively low income at SMEs. Based on the results, the job characteristics of a stratified labor market have a significant influence on life satisfaction regarding parenthood. To enhance parental life satisfaction and raise the fertility rate, the structure of the stratified labor market needs to be changed so that the labor market becomes more flexible and includes a solid social safety net.

## Introduction

Parents need a stable income and emotional stability to raise their children. According to OECD statistics, the fertility rate in Korea was 0.92 in 2019, exhibiting a steady decrease over the past 20 to 30 years (see Appendix A.1). The decreasing fertility rate raises questions about the common belief that parenthood is an important part of life that brings emotional rewards to parents. Parents' life satisfaction, the extent of enjoying a relatively flexible lifestyle and romance, declines with the birth of a child.

Glass (1) examined how parents' engagement in the labor market affected their life satisfaction after childbirth. The study found that parents experienced high stress levels from juggling parenthood and work because employment and child-rearing are fundamentally incompatible. Their increased stress levels result from a lack of flexible working hours, a shortage of high-quality childcare services for preschoolers, and inadequate policies and programs to reduce parental stress. The Korean labor market is stratified into sub-labor markets: large companies, small and medium-sized enterprises (SMEs), regular employees, and irregular employees. Sub-labor markets exist in a hierarchy with limited transferability between the markets, and the wages and working conditions among the markets continue to widen. Doeringer and Piore (2) argue that the labor market is divided into primary and secondary markets. Primary market jobs are “good jobs,” characterized by high wages, job security, and favorable working conditions. Meanwhile, secondary labor market jobs are characterized by low wages and relatively poor working conditions. Although there are no universal standards for defining Korea's primary and secondary labor markets, many researchers have divided the country's labor market based on firm size (large companies vs. SMEs) and employment type (regular employees vs. irregular employees) (3, 4).

Between the 1960s and the 1990s, large companies laid the foundation for export-led economic growth. Rapidly growing companies needed skilled employees and ensured high wages, employment benefits, and job security. Such conditions helped form Korea's internal labor market. However, changes occurred after the 1997 Asian International Monetary Fund financial crisis. As a result, Korean companies had to restructure and needed a flexible labor market to reduce labor costs (5). Following the crisis, the proportion of regular full-time workers decreased, whereas irregular workers, such as temporary and daily workers, increased (6, 7). Furthermore, the discrepancy in working conditions between internal and external labor markets widened. The widening gap likely increased income inequality and influenced life. Suppose the structure of such a stratified labor market does not improve. In that case, it will be difficult to expect young people to make positive decisions regarding marriage, childbirth, and parenting, all of which require money and time. In the long run, this will further lower the fertility rate, making it difficult to secure the necessary workforce, hindering Korea's growth potential.

According to Lombardo et al. (8), life satisfaction is strongly associated with self-reported mental health even after considering income, general health, and gender. Given this background, this study analyzes the effect of parenthood on life satisfaction using data from the first to twentieth Korean Labor and Income Panel Study. Section Literature Review presents the research data and the methodology used. Section Data and Methods presents the analysis results. Finally, Section Results presents the conclusions and implications.

## Literature Review

### South Korea's Stratified Labor Market

South Korea achieved rapid economic growth. For instance, its Gross domestic product (GDP) increased from 2,416.3 dollars in 1980 to 43,143.1 dollars in 2019.^1^ South Korea has the 4th largest economy by GDP in Asia and the 10th largest in the world. In addition, the rate of women's participation in economic activities increased from 47.6 percent in 1980 to 66.6% in 2017. However, as the rate of women's participation in economic activities increased, the fertility rate decreased from 2.8 to 1.1 in the same period, and it is still decreasing and showing no indications of rebounding (Appendix.A.2). What is causing South Korea's fertility rate to decrease continuously? First, South Korean women in their thirties as a child-rearing generation experienced the career discontinuity (7, 9). The social characteristics, based on South Korea's Confucian culture, includes a tradition that emphasizes women's roles in housework and infant care. Additionally, the male-centered organizational culture makes women's labor participation difficult to maintain. However, it is not only a problem for South Korean women. As the labor market experienced issues such as the decrease in youth employment and stagnation in the manufacturing industry, where the workers are mostly in their forties, both men and women are facing difficulties. As a reflection of this environment, young people, who must prepare for marriage, childbirth, and rearing, is investing more in education in order to increase their human capital. The government adopted policies and affirmative actions to maintain a balance between work and family, but there is no discernable effect (9).

In addition, the educational level of women in South Korea is not much different from that of men, and the rate of participation in economic activities has been increasing. Hence, the structure of the labor market in South Korea is an important case to consider. The size of a firm and permanent work status play an important role in determining the work conditions. As South Korea's economy grew rapidly between 1960 and 1990, workers in large firms have been able to secure higher wages, greater welfare benefits, and employment security. However, due to International Monetary Fund (IMF) requirements after the financial crisis in 1997, firms implemented structural reforms that would help them fight the economic crisis. Since then, the labor market developed a dual structure, with both permanent and temporary positions.

The Korean labor market is not a single labor market: it is a stratified labor market in terms of firm size (large or medium and small firms), work status (permanent or temporary positions), wage level, employment security, and welfare benefits for workers (5, 7). According to existing literature, the labor market in South Korea is divided into the primary labor market and secondary labor market. The primary labor market provides high wages, employment security, and sufficient opportunity for promotion. On the other hand, the secondary labor market provides low wages and has a high proportion of workers with temporary work status. Its characteristics also include poor working conditions and lack of promotion opportunities. According to Hwang (10), the proportion of workers in the primary labor market is 21.9% when using the firm's size and workers' permanent work status as direct indices to classify the primary and secondary labor markets. Various factors contribute to labor market segmentation, but it is believed that the industrial structure of the heavy chemical industry centered on the large firms that led the rapid economic growth in South Korea, created the dual structure of labor market. On the other hand, the policy response corresponding to firm size in Korea's labor market segmentation is also emphasized by permanent work status; namely, the segmentation by employment type or the relative effect of firm size (4).

### Parenthood and Life Satisfaction

Rising economic insecurity, inequalities, and the diffusion of intensive parenting ideology were major social contexts of parenting in the 2010s (11). Parents experience a gap between the ideal family and tough reality because the financial burden of raising children increases, leading to more stress and restrictions on the use of time.

(12) showed that the negative effect of parenthood on wellbeing could be explained by a large adverse impact on financial satisfaction, which dominates the positive impact on non-financial satisfaction. Walsh and Murphy (13) argued that any life satisfaction benefit derived from having children appears to be eroded for working parents. There is a negative association between life satisfaction for working mothers with children aged between 5 and 12 years. Furthermore, when both parents are working, the mothers' life satisfaction is also significantly reduced. (14) also indicated that children's effects on life satisfaction are negative for full-time parents.

Meanwhile, Baetschmann et al. (15) analyzed the relationship between parenthood and life satisfaction using cases in Germany. After controlling for household income, time, and health, they found that being a mother has a significant positive correlation with life satisfaction. (16) also found that happiness has a significant positive correlation with raising children. The correlation depended on having a mother, a partner, income level, and employment status. Roeters et al. (17) demonstrated that the beneficial effect of parenthood on individual wellbeing is determined by leisure and paid work status during the transition to parenthood. If fathers have numerous pre-birth levels of leisure activities, they experience a significant reduction in wellbeing from parenthood. (18)] found that the types of leisure for men and women increase at different rates, depending on whether they are parents. If both parents work for a living, then the mother's leisure time with the family increases, while the father has relatively more independent leisure time. Roeters et al. (17) showed that mothers working long hours experienced a reduction in the beneficial effect of parenthood. (19) revealed that work–family conflict is an important factor influencing parents' wellbeing in parenthood. In other words, the negative (or positive) effect of children on parents' subjective wellbeing is caused by the tension (reconciliation) between work and family. In particular, when a mother experiences work and family conflict, children experience a substantial negative effect on wellbeing. Glass (1) found that it is difficult for employment and child-rearing to coexist and that the stress of parenthood and participating in the labor market is high. Other reasons for this stress include a lack of policies and programs to reduce stress, such as the absence of flexible working hours and high-quality childcare services for preschoolers (1). Bonet et al. (20) stated that self-employed parents have a higher probability of childbirth than those with organizational employment. This is because work-family conflicts are larger for employees with permanent contracts than those under temporary contracts due to the dual job protection system. There is a higher probability of shifting to self-employment for women with temporary contracts before giving birth. (21) analyzed couples' dynamics in the employment–fertility link. Using cases in Belgium, they studied the relative incomes, job stability, time availability, and employment-sector-specific flexibility of dual earners regarding work regimes in the transition to parenthood. The results showed that women's greater time availability or access to flexible work regimes increased the probability of the first childbirth. Additionally, Lombardo et al. (8) showed that life satisfaction is strongly associated with self-reported mental health, even after considering income, general health, and gender.

Based on the above research trend, this study analyzes the effect of parenthood on life satisfaction in South Korea by reflecting on Korea's dual-labor market characteristics and draws implications for policies to promote a rebound in the fertility rate.

## Data and Methods

### Data

This study uses the first to twentieth Korean Labor and Income Panel Study, an annual longitudinal survey that tracks economic activity, labor market movements, income activities, and social life activities. Data on the variables for childbirth and household income were extracted from household surveys. Household income represents the sum of earned, financial, and real estate incomes from the previous year. Meanwhile, the attributes of individual respondents, such as life satisfaction, gender, age, marital status, as well as job characteristics such as wage, work experience, firm size, industry, and occupation, were obtained from personal surveys. Using panel data has an advantage as it enables us to control for unobservable individual effects.

**Figure 1:**
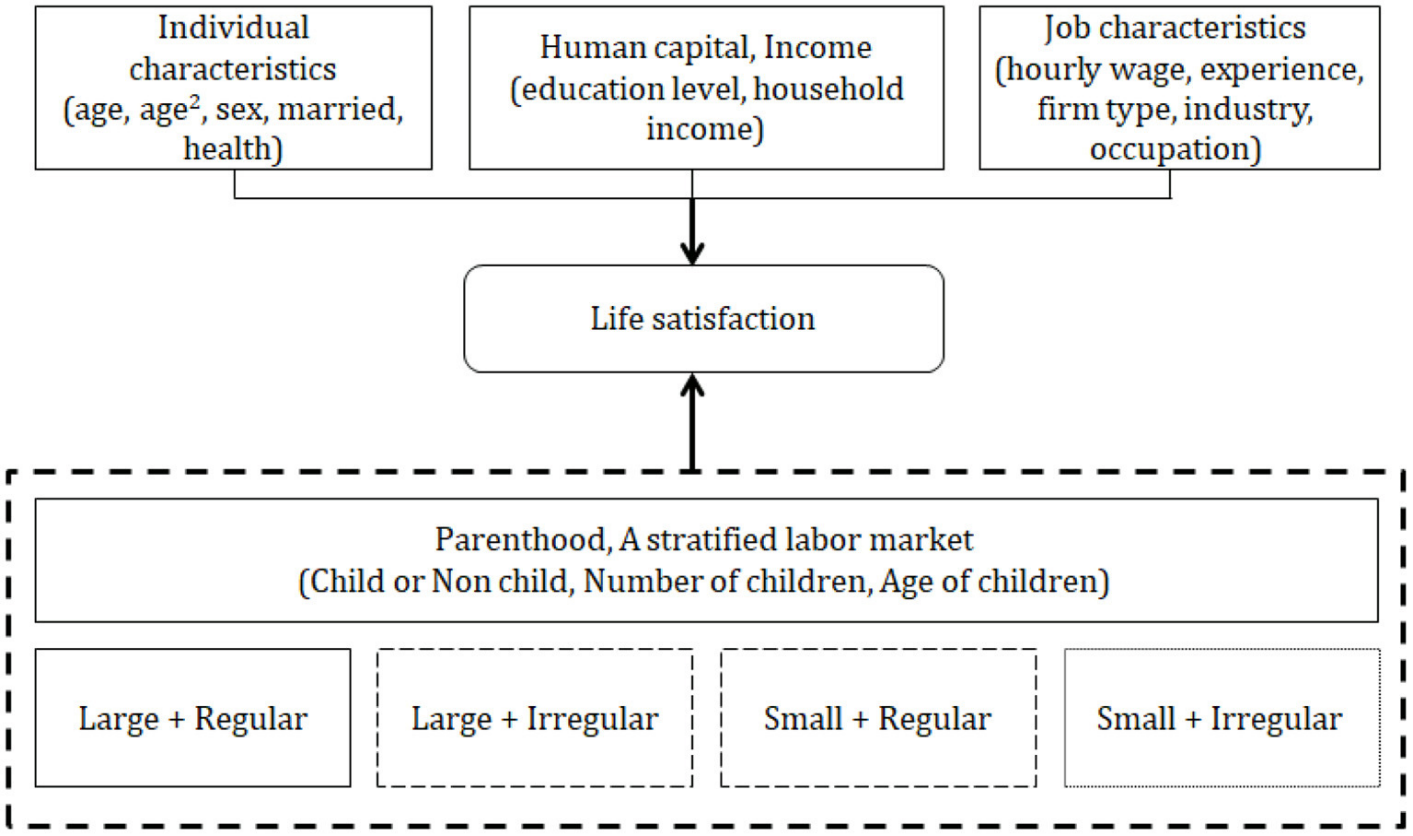
Conceptual framework.

The dependent variable is life satisfaction. As shown in Figure 1, the effects of parenthood, number of children, and age of children on parents' life satisfaction were analyzed by considering the different attributes in terms of individual characteristics, human capital and income, labor market participation, and job characteristics. From a total of 21,946 observations, the number of large companies with 300 or more employees was 6,733, whereas the number of SMEs with fewer than 300 workers was 15,213. The proportion of SMEs was larger than that of large companies. When the data were categorized by firm size and employment type, the largest category was regular employment at SMEs (hereinafter, SR), followed by regular employment at large companies (LR), irregular employment at SMEs (SI), and irregular employment at large companies (LI). In addition, LR and SR had a large proportion of men, whereas LI and SI had a large proportion of women.

Based on firm size and employment type (2 x 2), the respondents were divided into eight groups by parenthood [i.e., having a child (child) or not having one (non-child)], as shown in the bottom half of Table 1. Life satisfaction was measured on a 5-point scale, and the regular employment at large companies (LR) group showed the highest life satisfaction score (mean = 3.68). The average of life satisfaction score gradually decreased as we neared the peripheral labor market [LR(3.68) → LI(3.42) → SR(3.48) → SI(3.27)] In addition, the average of life satisfaction score in the SMEs-Regular group was higher than that of the large-irregular group.

**Table 1:** Basic statistics.

**Firm size**		**Large**				**Small**			
**(Large or small)**		**(*****N*** **=** **6,733)**				**(*****N*** **=** **15,213)**			
**Employment type**		**Regular**		**Irregular**		**Regular**		**Irregular**	
**(Regular or irregular)**									
**Obs**	Total	5,997		736		11,937		3,276	
	Male	4,629		269		7,887		1,168	
	Female	1,368		467		4,050		2,108	
	Ratio (F/M)	30%		174%		51%		180%	
**Parenthood** (**Child** **or Non-child)**		**Non-child [(1) LRN]**	**Child** **[(2) LRC]**	**Non-child [(3) LIN]**	**Child** **[(4) LIC]**	**Non-child [(5) SRN]**	**Child** **[(6) SRC]**	**Non-child [(7) SIN]**	**Child** **[(8) SIC]**
**Dependent** **Variable:**	Obs	859	5,138	116	620	2,009	9,928	621	2,655
Mean (%)	3.73 (100%)	3.67 (98%)	3.40 (91%)	3.42 (92%)	3.48 (93%)	3.48 (93%)	3.28 (88%)	3.27 (88%)
Life Satisfaction	Male	3.75	3.68	3.42	3.46	3.52	3.49	3.31	3.27
Female	3.69	3.63	3.38	3.39	3.43	3.47	3.26	3.28

*Source: korean labor and income panel study (1st−20th). Large firm(≥300 employees), Small firm(<300 employees)*.

### Methods

A fixed effects model was used to analyze the effects of parenthood and job characteristics on the parental life satisfaction. The fixed effect model is that the time-invariant characteristics are unique to the individual and should not be correlated with other individual characteristics. If the error terms are correlated, then the fixed effect is not suitable. To decide between fixed or random effects, we had run a Hausman test. It tests whether the unique errors are correlated with the regressors, the null hypothesis is they are not (22). In the Hausman test, the null hypothesis is rejected. So in this analysis, fixed effects estimator is consistent. Unobserved factors influencing parenthood can affect life satisfaction, and the method of ordinary least squares will likely lead to biased estimates because it does not consider such unobserved factors. Bias can be reduced by using a fixed effects model because it can solve the problem of heteroscedasticity, which does not change with time. In other words, it is possible to analyze the extent to which the value of the dependent variable changes when the explanatory variable changes by one unit for a certain individual. In addition, both observed and unobserved factors can be controlled (23).


(1) $$\begin{array}{r} {\text{Lifesatis}}_{\text{it}}{=\beta }_{\text{1}}\text{child}{\text{stratified}}_{\text{it}}^{\text{1}}+\beta_{\text{2}}\text{child}{\text{stratified}}_{\text{it}}^{\text{2}}\text{+}\beta_{\text{3}}{\text{indiv}}_{\text{it}}\\ \;+\beta_{\text{4}}{\text{jobcha}}_{\text{it}}\text{+}\alpha_{\text{i}}\text{+}{\text{u}}_{\text{it}} \end{array}$$

Equation (1) shows the effects of parenthood on life satisfaction. β_2_−β_1_ represent the incremental effect between different levels of the stratified labor market. The dependent variable is life satisfaction and *childstratified*_*it*_ are stratified labor market variables. Individual characteristics (age, married statue, education level etc.) and job characteristics (wage, experience, firm type etc.) are the control variables denoted as *indiv*_*it*_ and *jobcha*_*it*_ respectively. α_*i*_ is the unknown intercept for each individuals. *u*_*it*_ is the error term. The incremental effect can be used to compare life satisfaction following the birth of a child between different levels of the stratified labor market. Also, for this analysis, the STATA 15.0 MP version program was used.

## Results

The effects of parenthood on life satisfaction for different groups in the stratified labor market are shown in Table 2. The results for the eight groups, LRN^2^ ((1))–SIC ((8)), are presented in Table 1, and are defined by the labor market group the workers belong to and the alternating condition of child vs. no child. Model (1) shows the raw effects; Model (2) incorporates the terms of Model (1) plus individual characteristics (age/marriage/education/household income and health); and Model (3) incorporates the terms of Model (2) plus job characteristics (wage, experience, company type, occupation, and industry). The criterion variable is (8) SIC, which comprises small and irregular group parents with children.

**Table 2:** Effects of the parenthood on life satisfaction by stratified labor market group (FE).

	**Model 1**		**Model 2**		**Model 3**	
	**β**	**s.e**.	**β**	**s.e**.	**β**	**s.e**.
(1) Large·regular·no child (ref: SME·irregular·child)	0.115^***^	(0.025)	0.174^***^	(0.028)	0.172^***^	(0.030)
(2) Large·regular·child	0.049^***^	(0.018)	0.079^***^	(0.020)	0.083^***^	(0.022)
(3) Large·irregular·no child	0.032	(0.054)	0.057	(0.058)	0.108^*^	(0.064)
(4) Large·irregular·child	0.053^**^	(0.026)	0.046^*^	(0.027)	0.056^*^	(0.030)
(5) SME·regular·no child	0.080^***^	(0.019)	0.116^***^	(0.022)	0.121^***^	(0.025)
(6) SME·regular·child	0.050^***^	(0.014)	0.053^***^	(0.016)	0.055^***^	(0.018)
(7) SME·irregular·no child	0.088^***^	(0.027)	0.116^***^	(0.029)	0.135^***^	(0.034)
Age			0.010	(0.012)	−0.018	(0.013)
Age^2^			0.000	(0.000)	0.000	(0.000)
Separated (ref: Married)			−0.225^***^	(0.058)	−0.189^***^	(0.065)
Divorced			−0.211^***^	(0.033)	−0.206^***^	(0.037)
Widowed			−0.310^***^	(0.100)	−0.285^**^	(0.127)
Subjective health condition			0.194^***^	(0.006)	0.193^***^	(0.007)
Middle school graduate (ref: Did not graduate from middle school)			0.263	(0.202)	0.261	(0.199)
High school graduate			0.427^*^	(0.241)	0.282	(0.244)
Graduate of 2-year college			0.375	(0.245)	0.220	(0.250)
Graduate of 4-year college			0.335	(0.247)	0.178	(0.253)
Graduate of graduate school			0.264	(0.252)	0.099	(0.258)
Household income			0.022^***^	(0.005)	0.012^**^	(0.006)
Hourly wage					0.138^***^	(0.016)
Overtime work					−0.001^**^	(0.000)
Experience					0.001	(0.003)
Experience^2^					0.000	(0.000)
Public enterprise (ref: Private enterprise)					−0.016	(0.026)
Miscellaneous					0.090	(0.074)
Constant	3.377^***^	(0.012)	1.780^***^	(0.330)	1.757^***^	(0.431)
sigma_u	0.541		0.447		0.422	
sigma_e	0.496		0.455		0.448	
Rho	0.543		0.491		0.471	
N	31,422		24,865		21,946	
N of group	6,536		5,287		4,969	

*Source: Korean Labor and Income Panel Study (1st−20th)*.

* *p < 0.1*,

** *p < 0.05*,

*** *p < 0.01. sigma_u: standard deviation of residuals within groups, sigma_e: standard deviation of residuals (overall error term), Rho$={\left(sigma_{u}\right)}^{2}/{\left(sigma_{u}\right)}^{2}+{\left(sigma_{e}\right)}^{2}$*.

The absolute values of the coefficients increase from Model (1) to Model (3). The life satisfaction in most groups of Model 3, in which individual and job characteristics are incorporated, is higher than that of employees who have children and belong to the most peripheral labor market.

Taking Model (3) as the standard, life satisfaction for (1) LRN is 17.2% higher than that for (8) SIC. Life satisfaction for (2) LRC is 8.3% higher than that for (8) SIC. Life satisfaction following the birth of a child is lower in the peripheral labor market than in the core labor market. The incremental effect of parenthood is β_*LRC*_−β_*LRN*_ = 0.083−0.172 = −0.089, and parenthood lowers life satisfaction by 8.9% within LR. For workers with the same labor market characteristics, parenthood has a negative effect.

The life satisfaction of LRC is 5.6% higher than that of SIC. When comparing irregular workers, life satisfaction following the birth of a child is higher if the employee works for a large company as opposed to working at SMEs. The results are similar even when the firm size is the same but the employment type is different. Irrespective of firm size, life satisfaction following the birth of a child is higher among regular employees than among irregular employees (β_*LRC*_−β_*LIC*_ = 0.083−0.056 = 0.027, β_*SIC*_ = 0.055).

In the case of LR, life satisfaction following the birth of a child is significantly higher compared to SMEs employees, irregular employees, and SI. In the stratified labor market, there is a discrepancy between the core and peripheral labor markets in terms of the life satisfaction following childbirth and the type of labor market one belongs to. The latter affecting one's decision to have a child.

The effect of the birth of a child on life satisfaction for male and female workers by job characteristics is shown in Table 3. β_*m*_ < β_*f*_ for LRN compared to SIC, and life satisfaction is higher for a female worker than for a male worker when she is in the primary labor market and has no children. Further, life satisfaction is higher among female workers than male workers in the peripheral group. Analyzing the groups with children, the decrease in life satisfaction following the birth of a child is greater for women than for men; specifically, β_*LRC*_−β_*LRN*_ = 0.097−0.229 = −0.132 for women, whereas β_*LRC*_−β_*LRN*_ = 0.086−0.164 = −0.078 for men. For workers at SMEs, the decrease in life satisfaction following the birth of a child for women is greater than for men: β_*SRC*_−β_*SRN*_ = 0.045−0.140 = −0.095, whereas β_*SRC*_−β_*SRN*_ = 0.069−0.133 = −0.064 for men. An interpretation for this could the higher opportunity cost women in the labor market have due to childbirth. This opportunity cost is even higher for women working at large companies compared to those working at SMEs.

**Table 3:** Effects of parenthood on life satisfaction by stratified labor market group: Gender differences (FE).

	**Male**		**Female**	
	**β**	**s.e**.	**β**	**s.e**.
(1) Large ·regular·no child (ref: SME·irregular·child)	0.164^***^	(0.039)	0.229^***^	(0.051)
(2) Large·regular·child	0.086^***^	(0.030)	0.097^***^	(0.035)
(3) Large·irregular·no child	0.090	(0.089)	0.170^*^	(0.094)
(4) Large·irregular·child	0.065	(0.046)	0.052	(0.039)
(5) SME·regular·no child	0.133^***^	(0.034)	0.140^***^	(0.038)
(6) SME·regular ·child	0.069^***^	(0.026)	0.045^*^	(0.025)
(7) SME·irregular ·no child	0.185^***^	(0.055)	0.136^***^	(0.045)
Age	−0.029	(0.018)	0.010	(0.021)
Age^2^	0.000^*^	(0.000)	0.000	(0.000)
Separated (ref: Married)	−0.303^***^	(0.104)	−0.085	(0.084)
Divorced	−0.315^***^	(0.052)	−0.087^*^	(0.052)
Widowed	−0.088	(0.215)	−0.347^**^	(0.159)
Subjective health condition	0.195^***^	(0.008)	0.191^***^	(0.012)
Middle school graduate (ref: Did not graduate from middle school)	0.172	(0.219)	0.678	(0.472)
High school graduate	0.229	(0.289)	0.635	(0.520)
Graduate of 2-year college	0.195	(0.303)	0.567	(0.524)
Graduate of 4-year college	0.139	(0.305)	0.560	(0.527)
Graduate of graduate school	0.045	(0.311)	0.501	(0.540)
Household income	0.004	(0.007)	0.026^**^	(0.010)
Hourly wage	0.186^***^	(0.021)	0.067^***^	(0.026)
Overtime work	−0.002^***^	(0.001)	0.001	(0.001)
Experience	0.000	(0.003)	0.004	(0.005)
Experience^2^	0.000	(0.000)	0.000	(0.000)
Public enterprise (ref: Private enterprise)	0.012	(0.034)	−0.050	(0.039)
Miscellaneous	0.057	(0.119)	0.119	(0.094)
Constant	2.166^***^	(0.517)	1.623^**^	(0.735)
sigma_u	0.382		0.479	
sigma_e	0.446		0.450	
Rho	0.423		0.531	
N	13,953		7,993	
N of group	2,820		2,149	

*Source: Korean Labor and Income Panel Study(1st−20th)*.

* *p < 0.1*,

** *p < 0.05*,

*** *p < 0.01. sigma_u: standard deviation of residuals within groups, sigma_e: standard deviation of residuals (overall error term), Rho$={\left(sigma_{u}\right)}^{2}/{\left(sigma_{u}\right)}^{2}+{\left(sigma_{e}\right)}^{2}$*.

In the case of SI, the decrease in life satisfaction following the birth of a child is greater for men than for women (0.185 > 0.136). In Korea, jobs at SMEs are deemed inferior than jobs at large companies; moreover, there are fewer opportunities to enter large companies than SMEs. Due to the aforementioned reasons, (1) women working at large companies exhibit a greater decrease in life satisfaction following the birth of a child; (2) men working at SMEs exhibit a greater decrease in life satisfaction following the birth of a child, since men in Korea are generally the income earners in the family but wages and benefits are lower at SMEs than at large companies; and (3) women working at SMEs tend to experience a relatively slight decrease in life satisfaction following the birth of a child than women working at large companies because there are more opportunities for employment at SMEs than at large companies.

The results for the effect of having more children on parental life satisfaction showed that the life satisfaction for those with three children or more is higher for LR compared with that for SI. In terms of gender, the overall effect of the number of children and job characteristics on life satisfaction was not significant among women (Table 4). For men, however, employment type was an important factor affecting life satisfaction following the birth of children. Regardless of firm size, regular employees exhibited higher life satisfaction compared to those in SI. As the number of children increases, employment stability becomes a major factor affecting life satisfaction for men compared to women.

**Table 4:** Effect of the number of children on life satisfaction by stratified labor market group (FE).

	**Total**		**Male**		**Female**	
	**β**	**s.e**.	**β**	**s.e**.	**β**	**s.e**.
(1) Large·regular·1 child (ref: SME·irregular·3 children or more)	0.120^**^	(0.057)	0.200^**^	(0.082)	0.066	(0.086)
(2) Large·regular·2 children	0.081	(0.055)	0.153^*^	(0.080)	0.027	(0.081)
(3) Large·regular·3 children or more	0.105	(0.065)	0.178^**^	(0.087)	0.010	(0.124)
(4) Large·irregular·1 child	0.032	(0.072)	0.203^*^	(0.108)	−0.105	(0.100)
(5) Large·irregular·2 children	0.073	(0.062)	0.131	(0.095)	0.011	(0.085)
(6) Large·irregular·3 children or more	0.230^*^	(0.130)	0.177	(0.166)	0.393^*^	(0.221)
(7) SME·regular·1 child	0.070	(0.055)	0.165^**^	(0.081)	−0.021	(0.079)
(8) SME·regular·2 children	0.076	(0.053)	0.158^**^	(0.078)	−0.006	(0.075)
(9) SME·regular·3 children or more	0.094^*^	(0.055)	0.165^**^	(0.079)	0.031	(0.080)
(10) SME·irregular·1 child	−0.001	(0.058)	0.036	(0.088)	−0.041	(0.082)
(11) SME·irregular·2 children	0.023	(0.055)	0.117	(0.081)	−0.069	(0.076)
Age	−0.012	(0.016)	−0.015	(0.021)	0.002	(0.029)
Age^2^	0.000	(0.000)	0.000	(0.000)	0.000	(0.000)
Separated (ref: Married)	−0.207^**^	(0.085)	−0.210	(0.140)	−0.194^*^	(0.108)
Divorced	−0.183^***^	(0.052)	−0.292^***^	(0.075)	−0.071	(0.074)
Widowed	−0.252	(0.158)	−0.109	(0.237)	−0.386^*^	(0.217)
Subjective health condition	0.196^***^	(0.007)	0.197^***^	(0.009)	0.194^***^	(0.013)
Middle school graduate (ref: Did not graduate from middle school)	0.340	(0.212)	0.236	(0.236)	0.673	(0.476)
High school graduate	0.424	(0.272)	0.296	(0.303)	0.880	(0.619)
Graduate of 2-year college	0.363	(0.280)	0.231	(0.317)	0.841	(0.625)
Graduate of 4-year college	0.355	(0.284)	0.170	(0.320)	0.979	(0.634)
Graduate of graduate school	0.295	(0.290)	0.101	(0.327)	0.971	(0.649)
Household income	0.019^**^	(0.008)	0.007	(0.009)	0.042^***^	(0.015)
Hourly wage	0.133^***^	(0.018)	0.196^***^	(0.023)	0.036	(0.030)
Overtime work	−0.001	(0.001)	−0.002^***^	(0.001)	0.002^**^	(0.001)
Experience	0.003	(0.003)	0.003	(0.004)	0.004	(0.006)
Experience2	0.000	(0.000)	0.000	(0.000)	0.000	(0.000)
Public enterprise (ref: Private enterprise)	−0.011	(0.028)	0.030	(0.038)	−0.060	(0.043)
Miscellaneous	0.060	(0.084)	0.040	(0.131)	0.075	(0.110)
Constant	1.411^***^	(0.517)	1.724^***^	(0.603)	1.386	(0.896)
sigma_u	0.415		0.383		0.465	
sigma_e	0.448		0.446		0.453	
rho	0.461		0.424		0.513	
N	18,341		11,971		6,370	
N of group	4,171		2,445		1,726	

*Source: Korean Labor and Income Panel Study (1st−20th)*.

* *p < 0.1*,

** *p < 0.05*,

*** *p < 0.01. sigma_u: standard deviation of residuals within groups, sigma_e: standard deviation of residuals (overall error term), Rho$={\left(sigma_{u}\right)}^{2}/{\left(sigma_{u}\right)}^{2}+{\left(sigma_{e}\right)}^{2}$*.

The age of the child (children) is another important factor for the life satisfaction of parents participating in the labor market (24–26) (Table 5). This is because the younger the child, the more that caregivers need to devote to time and physical costs. In Korea, many women take career breaks when their children enter elementary schools. This is because a great deal of emphasis is placed on education during the elementary school years. Moreover, classes tend to end early in the day without providing childcare services for children who need parental care, unlike preschoolers who can be taken care of at daycare centers all day.

**Table 5:** Effects of children's age on life satisfaction by stratified labor market group (FE).

	**Total**		**Male**		**Female**	
	**β**	**s.e**.	**β**	**s.e**.	**β**	**s.e**.
(1) Large·regular·0–5 (ref: SME·irregular·13–18)	0.107^***^	(0.032)	0.110^***^	(0.041)	0.137^**^	(0.059)
(2) Large·regular·6–12	0.147^***^	(0.041)	0.159^***^	(0.051)	0.136^*^	(0.079)
(3) Large·regular·13–18	0.069^***^	(0.026)	0.064^*^	(0.036)	0.090^**^	(0.043)
(4) Large·irregular·0–5	0.105	(0.068)	0.113	(0.085)	0.100	(0.115)
(5) Large·irregular·6–12	0.060	(0.111)	0.279^*^	(0.162)	−0.150	(0.155)
(6) Large·irregular·13–18	0.042	(0.036)	0.038	(0.055)	0.052	(0.048)
(7) SME·regular·0–5	0.095^***^	(0.028)	0.096^**^	(0.037)	0.138^***^	(0.050)
(8) SME·regular·6–12	0.023	(0.031)	0.030	(0.042)	0.029	(0.050)
(9) SME·regular·13–18	0.063^***^	(0.022)	0.070^**^	(0.032)	0.054^*^	(0.031)
(10) SME·irregular·0–5	−0.079^*^	(0.044)	−0.053	(0.056)	−0.092	(0.072)
(11) SME·irregular·6–12	0.018	(0.050)	0.014	(0.068)	0.011	(0.074)
Age	−0.008	(0.017)	−0.013	(0.021)	0.018	(0.030)
Age^2^	0.000	(0.000)	0.000	(0.000)	0.000	(0.000)
Separated (ref: Married)	−0.117	(0.091)	−0.174	(0.147)	−0.066	(0.116)
Divorced	−0.210^***^	(0.057)	−0.254^***^	(0.078)	−0.142^*^	(0.085)
Widowed	−0.243	(0.211)	−0.142	(0.238)	−0.753	(0.470)
Subjective health condition	0.198^***^	(0.008)	0.200^***^	(0.009)	0.192^***^	(0.014)
Middle school graduate (ref: Did not graduate from middle school)	0.726^**^	(0.279)	0.744^**^	(0.345)	0.670	(0.475)
High school graduate	0.701^**^	(0.335)	0.680^*^	(0.405)	0.892	(0.618)
Graduate of 2-year college	0.653^*^	(0.342)	0.616	(0.416)	0.864	(0.626)
Graduate of 4-year college	0.649^*^	(0.345)	0.569	(0.418)	0.979	(0.635)
Graduate of graduate school	0.593^*^	(0.350)	0.502	(0.423)	0.946	(0.651)
Household income	0.022^***^	(0.008)	0.007	(0.009)	0.056^***^	(0.016)
Hourly wage	0.138^***^	(0.019)	0.198^***^	(0.023)	0.033	(0.031)
Overtime work	−0.001	(0.001)	−0.002^***^	(0.001)	0.002^**^	(0.001)
Experience	0.002	(0.003)	0.004	(0.004)	0.001	(0.006)
Experience^2^	0.000	(0.000)	0.000	(0.000)	0.000	(0.000)
Public enterprise (ref: Private enterprise)	−0.009	(0.029)	0.030	(0.038)	−0.060	(0.045)
Miscellaneous	0.061	(0.086)	0.039	(0.131)	0.081	(0.116)
Constant	1.061^*^	(0.559)	1.315^**^	(0.659)	0.961	(0.921)
sigma_u	0.418		0.388		0.459	
sigma_e	0.447		0.445		0.452	
Rho	0.466		0.433		0.508	
N	17,502		11,750		5,752	
N of group	4,023		2,404		1,619	

*Source: Korean Labor and Income Panel Study (1st−20th)*.

* *p < 0.1*,

** *p < 0.05*,

*** *p < 0.01*.

*sigma_u: standard deviation of residuals within groups, sigma_e: standard deviation of residuals (overall error term), Rho$={\left(sigma_{u}\right)}^{2}/{\left(sigma-u\right)}^{2}+{\left(sigma_{e}\right)}^{2}$*.

The analysis of the results are as follows: the life satisfaction for LR group is higher than that of the reference group regardless of the child's age. In addition, overall, the absolute value of the coefficient is higher for women, implying that working as a regular employee at a large company has a greater effect. The negative effect on life satisfaction is significant for SI, particularly when they have children aged below five. Furthermore, life satisfaction is higher among regular employees than irregular employees (i.e., the type of employment is important) regardless of firm size, with life satisfaction being higher for a regular employee with a child under five than for the reference group.

## Discussion

This study analyzes the effect of parenthood on life satisfaction in South Korea using stratified labor market data from the Korean Labor and Income Study. The results showed that parental life satisfaction was significantly lower for the irregular SME workers than regular large firm employees. Additionally, women participating in the labor market have a high opportunity cost of having children. In Korea, SME jobs are regarded as inferior to those in large companies, and there are fewer opportunities for entering large companies than in SMEs. Additionally, a woman's decrease in life satisfaction following childbirth is greater than a man's, particularly if she is a regular employee at a large company, owing to the higher opportunity cost in wages, employment maintenance, promotion, etc. In contrast, in the case of irregular SME workers, the decrease in parental life satisfaction was higher for men than for women. This is because men are generally the income earners in Korean families, and working conditions such as wages and benefits are not favorable for SMEs-irregular workers compared to regular employees at large companies.

Regarding the effect of the number of children, parental life satisfaction in parents with three or more children was higher for regular employees and those at large companies than for irregular and SME employees. The higher the number of children, the greater the effect of employment stability on life satisfaction for men compared with women. Preisner et al. (27) showed that the parenthood happiness gap between men and women decreased with the decline in gendered parenthood norms. However, this study verifies that Korea's stratified labor market characteristics affect parental life satisfaction. Suppose the labor market structure does not improve. In that case, the fundamental problem of the decreasing fertility rate will not be solved, even though the Korean government is implementing various measures to increase fertility rates, such as childcare allowances and parental leave policies. Therefore, to solve the vicious cycle of low fertility rates, the gap in working conditions between workers in large companies and SMEs must be reduced.

Furthermore, the labor market structure, which is stratified into regular and irregular employment, should be improved by providing equal pay for equal labor and ensuring employment stability. Women's opportunity costs of childbirth and child-rearing must be reduced to promote marriage and childbirth. Additionally, a wide range of employment opportunities should be offered according to the life cycle of the flexible labor market. It is difficult to expect a rebound in the birth rate unless the negative effects of childbirth on life satisfaction are addressed. A solid social safety net should also be established to prevent working parents from experiencing negative conditions, and because of the burden of balancing parenthood with work. This study analyzed the effect of parenthood by firm size and employment type on life satisfaction. There is a limitation in that self-employed or gig workers are not included. Since employment types are diversifying worldwide, the effect of parenthood on life satisfaction, reflecting the characteristics of low stability jobs, remains a topic for future research.

## Data Availability Statement

Publicly available datasets were analyzed in this study. This data can be found here: The datasets Korean Labor and Income Panel Study for this study can be found in the Korea Labor Institute website (http://www.kli.re.kr/klips/index.do).

## Author Contributions

JC contributed to conception and design of the study. HJ organized the database and performed the statistical analysis. All authors contributed to writing the first draft of the manuscript, revision, and approved the submitted version.

## Funding

This research was supported by SKKU Excellence in Research Award Research Fund, Sungkyunkwan University, 2021.

## Conflict of Interest

The authors declare that the research was conducted in the absence of any commercial or financial relationships that could be construed as a potential conflict of interest.

## Publisher's Note

All claims expressed in this article are solely those of the authors and do not necessarily represent those of their affiliated organizations, or those of the publisher, the editors and the reviewers. Any product that may be evaluated in this article, or claim that may be made by its manufacturer, is not guaranteed or endorsed by the publisher.

## Footnotes

^1^US dollars per capita (current PPP).

^2^Large·Regular·No child(LRN), Large·Regular·Child(LRC), Large·Irregular·No child(LIN), Large·Irregular·Child(LIC), SME·Regular·No child(SRN), SME·Regular·Child(SRC), SME·Irregular·No child(SIN), SME·Irregular·Child(SIC).
