# Supplementary material for: Parenthood and Life Satisfaction in Stratified Labor Market: Evidence From Korea
Source: Front Public Health. 2022 Jun 2;10:874877. doi: 10.3389/fpubh.2022.874877 (PMC9201438; doi:10.3389/fpubh.2022.874877)
Supplement: Supplementary file 1 [file Table_1.docx]

**Appendix.**

| [Appendix.1] Fertility rate in Organization for Economic Co-operation and Development (OECD) countries (2019). |
| --- |
| Source: OECD database (https://data.oecd.org/pop/fertility-rates.htm). 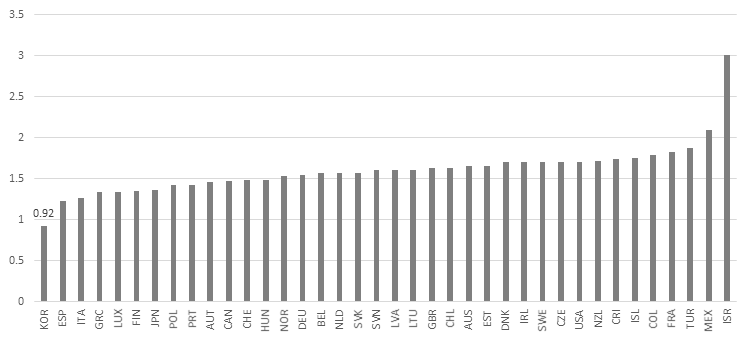 |
| \| [Appendix.2] Change in fertility rate and economic activity participation rate of women in South Korea  (Unit: %) \| \| --- \| \|  \| |

Source: OECD database.
